# AUTOMATIC QUANTIFICATION OF TANDEM WALKING USING A WEARABLE DEVICE: VALIDITY OF THE INSTRUMENTED TANDEM WALK

**DOI:** 10.1093/geroni/igz038.1217

**Published:** 2019-11-08

**Authors:** Jeffrey M Hausdorff, Natalie Ganz, Eran Gazit, Amit Hadad, Aron S Buchman, Anat Mirelman

**Affiliations:** 1 Center for the study of Movement, Cognition and Mobility, Neurological Institute, Tel Aviv Sourasky Medical Center, Israel, Tel Aviv, Israel; 2 Department of Physical Therapy, Sackler Faculty of Medicine, Tel Aviv University, Israel, Tel Aviv, Israel; 3 Rush Alzheimer’s Disease Center, Rush University Medical Center, Chicago, USA, Chicago, Illinois, United States

## Abstract

Tandem walk (TW) is typically assessed by the time to complete the task and the number of missteps, however, these measures suffer from limitations and may not fully capture the range of performance in this task. We developed metrics of TW by using a body-fixed, wearable sensor in young and older adults. Healthy young men (n=40) and older adult men (n=362) were studied. While wearing a 3D accelerometer on their lower back, subjects performed three different tasks: TW, usual-walking, and quiet standing. The extracted measures for TW were: High-to-Low frequency band ratio from the power spectral density from the ML axis [nu], signal vector magnitude[g], step duration[s], sample entropy from ML, AP axis[nu] and CV[%]. All of the TW metrics were significantly different in the young and older men (p<0.001). Older men completed the TW with higher CV, suggesting greater stride-to-stride variability and they walked more slowly, as seen by their step duration. Additionally, the frequency ratio measure suggests that the older adults displayed less complex corrective movements in the ML axis. TW measures were modestly correlated with usual-walking (e.g., average stride time with TW step time, r=0.3; p<0.001) and with quiet standing postural control (e.g., acceleration path length in the ML and AP axis with TW sample entropy in the ML axis, r=0.13; p=0.014). Metrics derived from a wearable device complement conventional TW measures and vary with age. Further work is needed to determine if TW, gait and posture metrics are differentially associated with distinct adverse health outcomes.

